# Treatment of hydatid cyst and bilio-bronchial and bilio-pleuro-bronchial fistulas via thoracotomy and transdiafragmal approach from October 2005 to October 2012 in a single unit in Tirana, Albania

**DOI:** 10.1186/1749-8090-8-S1-O257

**Published:** 2013-09-11

**Authors:** F Gradica, A Menzelxhiu, F Kokiqi, E Fype, A Cami, E Shehu, D Argjiri, P Kapisyzi, I Skenduli, F Caushi

**Affiliations:** 1 Thorax Surgery Department, University Hosptal "Shefqet Ndroqi", Tirana, Albania; 2 Anesthesiology and Reanimation Department, University Hosptal "Shefqet Ndroqi", Tirana, Albania; 3 Pneumology Department, University Hosptal "Shefqet Ndroqi", Tirana, Albania

## Background

The aim of this study was to report the results of surgical treatment of hydatid bilio-bronchial et bilio-pleuro-bronchial fistulas via thoracotomy and transdiafragmal approach.

## Methods

From October 2005 to October 2012, 13 cases were observed in the same center. Biliptysis was the main symptom in 80% of cases. The diagnosis was based on chest radiography ,thoracic and abdominal CT and abdominal ultrasonography, fibrobronchoscopy; all examinations visualised the cyst, intrathoracic collections, a diaphragmatic breach and biliary lesions. All patients were treated by one-stage thoracotomy. The procedures consisted of lung resection (lobectomy and/or segmentectomy) (n=11) and decortication (n=6) in the chest, cyst dome resection (n=13) or part pericystectomy (n=2) in the abdomen and suture and plastic of the diaphragmatic defect in all cases after hepato-diaphragmatic deconnection. An additional laparotomy was not necessary in all cases.

## Results

There were 2 deaths (15.3%):no one intraoperative death and two postoperative deaths, mostly related to pulmonary complications. Postoperative complications (14.3%) were mainly respiratory. Clinical and radiological results were good with a one-year follow-up.

## Conclusions

Bilio-bronchial and bilio-pleurobronchial fistulas due to hydatid cyst are rare, but severe diseases. They are responsible for lesions at three levels: abdominal, diaphragmatic and thoracic. A high perioperative mortality rate was observed. Thoracotomy with transdiafragmal approach is the best approach for surgical treatment at all three levels.

